# Microbiome profiling of rotavirus infected children suffering from acute gastroenteritis

**DOI:** 10.1186/s13099-021-00411-x

**Published:** 2021-03-29

**Authors:** Muhammad U. Sohail, Hebah A. Al Khatib, Asmaa A. Al Thani, Khalid Al Ansari, Hadi M. Yassine, Maha Al-Asmakh

**Affiliations:** 1 Proteomics Core, Weill Cornell Medicine-Qatar, P.O. Box 24811, Doha, Qatar; 2 Biomedical Research Center, Qatar University, P.O. Box 2713, Doha, Qatar; 3 Department of Biomedical Sciences, College of Health Sciences, QU Health, Qatar University, P.O. Box 2713, Doha, Qatar; 4 Emergency Medicine Department, Sidra Medicine, Qatar Foundation, Doha, Qatar; 5 Biomedical and Pharmaceutical Research Unit, QU Health, Qatar University, P.O. Box 2713, Doha, Qatar

**Keywords:** Rotavirus, Rotavirus vaccine, Acute gastroenteritis, Microbiome, Proteobacteria

## Abstract

**Background:**

Rotavirus (RV) is a leading cause of pediatric diarrhea and mortality worldwide. The virus causes acute gastroenteritis characterized by moderate to severe vomiting, diarrhea, dehydration, and fever. Microbial dysbiosis caused by RV infection may significantly influence disease prognosis and the development of other chronic diseases. The gut microbiome plays a vital role in enteric immune response for rotavirus vaccine (RVV) that requires further elucidations. The current study evaluates the gut microbiome of RV positive children and compares gastroenteritis manifestation in children admitted to the Pediatric Emergency Centre, Hamad Medical Cooperation, Doha, Qatar. Stool samples were collected from thirty-nine RV positive and eight healthy control children. 16S rRNA sequence was performed using the Illumina MiSeq platform.

**Results:**

The data demonstrated a significant increase in microbiome diversity denoted by higher relative abundances of phylum Proteobacteria (p = 0.031), Fusobacteria (p = 0.044) and genus *Streptococcus* (p ≤ 0.001) in the infected group relative to the control. Similarly, district clustering pattern (PERMANOVA p = 0.01) and higher species richness (Shannon entropy p = 0.018) were observed in the children who received two RVV doses compared with the non-vaccinated or single-dose groups. These microbiome changes were represented by over-abundance of phylum Bacteroidetes (p = 0.003) and Verrucomicrobia (p ≤ 0.001), and lower expression of family *Enterobacteriaceae* in two RVV doses group. However, microbiome composition was not associated with diarrhea, vomiting, and other parameters of gastroenteritis.

**Conclusions:**

The observations assert significant microbial signatures of RVV, which is dose-dependent, and suggest manipulating these microbes as a novel approach for improving RVV efficacy. Further studies are warranted to investigate the immune status of these patients and mechanistic investigation to enhance RVV seroconversion.

## Background

Diarrhea is the second leading cause of mortality in children after pneumonia, causing one out of nine child deaths worldwide [1]. The Global Burden of Disease Study 2016 estimated that rotavirus (RV) caused 128,500 deaths in children and was responsible for an estimated 258 million infectious diarrhea cases worldwide [1]. Despite the availability of the rotavirus vaccine (RVV), the virus is the leading cause of diarrhea-related mortalities in children under five years of age [2]. Literature shows that vaccine efficacy is region-specific and depicts poor seroconversion, particularly in low-and middle-income countries [2]. Human clinical trial data suggest a possible link between the gut microbiome and the enteric immune system’s response for low RVV efficacy [2–4].

The human gut is occupied by a large and complex microbial community that maintains the physiological homeostasis of the host immune and neuroendocrine systems. The gastrointestinal microbiome is nvolved in the digestion and absorption of nutrients and is essiential for priming the gut-associated lymphoid tissue (GALT) that helps inhibit local or systemic infections. Therefore, many gut realted disorders including viral infections have been associated with changes in the microbiome [5, 6].

The gut microbiome has been implicated in the pathogenesis of several viral pathogens, including adenovirus, astrovirus, calicivirus, coronavirus, norovirus, poliovirus, and RV [7, 8]. Furthermore, mounting evidence also supports the role of gut microbiome in determining immunogenicity and seroconversion of the oral RVV [2, 3, 6]. Germ-free murine studies have shown that normal GALT development requires bacterial colonization of the gut with specific microbes that increase IgA secretion and enhance RVV immunogenicity [9]. Murine studies also suggest that the gut microbiome can help prevent and cure RV infection and serve as an adjuvant in RVV vaccination, as demonstrated by the ability of segmented filamentous bacteria to cure RV by increasing epithelial cell turnover and antibody response [10].

Compared to the healthy controls, children infected with RV have reduced microbiome diversity, and antibiotic treatment further exacerbates microbiome diversity and disease outcomes [3, 6]. Nevertheless, the available literature on the involvement of gut microbiome in the pathogenesis of RV infection is still scarce, underlining the need for further investigation. Therefore, the present study investigates gut microbiome composition in children suffering from RV infection and correlates microbiome composition and diversity with gastroenteritis.‎

## Materials and methods

### Patient sampling

Stool samples were collected from children affected by acute gastroenteritis (AGE) during their visit to the Pediatric Emergency Centre (PEC), HMC, Doha, Qatar. All collected samples were screened using the FilmArray Gastrointestinal (GI) Panel Kit® (BioFire Diagnostics, USA) for viral and bacterial infections. Multiple clinical characteristics of AGE were present in the admitted children, including fever, diarrhea, vomiting, and dehydration. Vesikari score system was used to evaluate the severity of AGE of the participants [11]. Similarly, children were also categorized based on their vomiting and diarrhea indexes. The vomiting and diarrhea indexes were calculated by multiplying their frequencies with durations in days (vomiting/diarrhea frequency × duration in days). Stool samples were collected before the administration of any medications. Vomiting, diarrhea indexes, and other demographic information are presented in Table 1.

**Table 1:** Demography and clinical characteristics of the enrolled children

Demography	Rotavirus positive	Control
Number of children	39	8
Age in months median (interquartile range; IQR)	20 (36.5)	13.5 (14.5)
Gender (M, F)	23.16	5.3
Rotavirus genotype		
G1P8	6 (15.3%)	NA
G2P4	4 (10.3%)	
G3P4	2 (5.1 %)	
G3P8	25 (64.1%)	
G9P8	2 (5.1 %)	
Rotavirus vaccination status		
Non-vaccinated	21 (53.8%)	NA
1_dose	11 (28.2)	
2_doses	7 (17.9)	
Vesikari index		
Sever	31 (71.4%)	NA
Moderate	8 (20.5%)	
Mild	0 (0%)	
Diarrhea		
Frequency median (IQR)	6 (3)	NA
Duration in days Median (IQR)	2 (2)	
Diarrhea index (frequency × duration in days)		
Mild (≤ 8)	16 (41%)	
Moderate (9 to 15)	13 (33.3%)	
Severe (≥ 16)	10 (25.6%)	
Vomit		
Frequency median (IQR)	5 (3.75)	NA
Duration in days Median (IQR)	2 (2)	
Vomiting index (frequency × duration in days)		
Mild (≤ 8)	15 (38.4%)	
Moderate (9 to 15)	11 (28.2%)	
Severe (≥ 16)	13 (33.3%)	
Hospitalization	34 (87.1%)	
Fever	1 (2.5%)	NA
Dehydration		
Mild	18 (46.1%)	
Moderate	21 (53.8%)	

### Amplicon sequencing

Genomic DNA was extracted using QIAamp DNA Mini Stool Kit (cat. no. 51,504; Qiagen, Hilden, Germany). DNA quality control (QC) was checked using NanoDrop-2000 (Thermo Fisher Scientific, USA), Qubit-4 (Life Technologies, USA), and Agilent bioanalyzer 2100 (Agilent Technologies, US). DNA libraries were prepared using 337F/805R primer-pair targeting V3-V4 region of the 16S rRNA gene. All libraries were dual indexed using Illumina Nextera XT Library Prep. Kit (FC-131-1002, Illumina Inc., USA). Amplicon libraries were normalized using Agencourt AMpure XP beads (Beckman Coulter, USA). All libraries were pooled together for performing sequencing using MiSeq v3 kit (MS-102-3003; Illumina Inc., USA). MiSeq run generated 300 bp long paired-end reads implemented as FASTQ files.

### Data analysis

MiSeq data were analyzed using QIIME2 software. In brief, FASTQ files were imported into QIIME2 artifacts. Sequence QC was performed using DADA2 plugin to generate FeatureTable [Frequency] and FeatureData [Sequence]. Phylogenetic diversity analysis was performed using mafft-fasttree plugin. Greengenes 13 − 8 database was used for taxonomic analysis in q2-feature-classifier. Alpha and beta-diversity analysis was performed at 13,813 bp sampling depth using Shannon and weighted_unifrac indexes, respectively. Taxonomic description for microbiome was only represented at phylum and species/genus level. Statistical analysis was performed on only those phyla and species/genera with a relative abundance of at least 0.5 % or was present in at least 50 % of the samples, and the remaining bacterial clades were discarded.

### Statistical analysis

The participants were classified into multiple categorical groups based on their history of infection (healthy and sick), vaccine history, the RV genotype, gender, age, and severity index of vesicular gastroenteritis. ‎Non-parametric Mann-Whitney U or Kruskal Wallis tests were conducted to compare microbiome taxonomy and diversity among different categorical groups. ‎Principal coordinates (PCoA) plots were constructed for beta diversity analysis, and statistical analyses were performed using pairwise PERMANOVA test. The association of microbiome and medical history was evaluated using Spearman’s rank correlation coefficient test.

## Results

### Study design and population demography

A total of forty-seven stool samples, including 39 from children (median age 20 months) with RV infection and eight samples from healthy children (median age 13.5 months), were analyzed (Table 1). Children were categorized as infants (≥ 12 months; n = 13), toddlers (12 to 36 months; n = 23), and pre-School (≤ 36 month; n = 11). The most prevalent RV genotype was G3P8 (n = 25), followed by G1P8 (n = 6), G2P4 (n = 4), G3P4 (n = 2), and G9P8 (n = 2). Of the 39 infected children, 31 children had severe gastrointestinal symptoms (Vesikari score ≥ 11), and only 8 had moderate symptoms (Vesikari score ≤ 10). In the infected group, 11 children were one-dose RVV (1_dose), seven were vaccinated with two RVV doses (2_doses), and the remaining 21 were non-vaccinated (0_dose).

### Microbiome community composition

For forty-seven samples, 4,289,304 (median 87,143; range 11,517 to 244,265) raw reads were produced. ‎Raw reads were subjected to QC before further analysis. Sequence pairing, denoising, and elimination of chimeras were involved in the QC filtration step, which yielded 1,011,316 (median 20,443; range 10,057 to 40,913) good quality reads. There were 5,775 features (OTUs) in the final dataset with a frequency range of one to a maximum of 37,751. These high-quality features were classified against the Greengenes reference database for taxonomic analysis. Taxonomic analysis of forty-seven samples classified OTUs into 64 phyla (4 unassigned), 177 classes (45 unassigned) 331 orders (117 unassigned), 469 families (254 unassigned), and 707 genera (422 unassigned), and 837 species (632 unassigned). To provide correct taxonomic classification, less than 25% of the 16S rRNA samples resolved to species level, and the remaining features could not be classified accurately. In descending order, the four most dominant phyla present in the microbiome of the study population were Firmicutes (45.6%), Proteobacteria (21.2%), Actinobacteria (12.5%), and Bacteroidetes (10.7%). Similarly, the highest species diversity was observed in phylum Firmicutes (80 bacterial species), followed by Proteobacteria (57), Bacteroidetes (28), and Actinobacteria (19). For statistical analysis, twelve most abundant bacterial genera/species were used to compare taxonomic differences among studied groups.

### Microbiome comparison between infected and healthy children

**Fig. 1:**
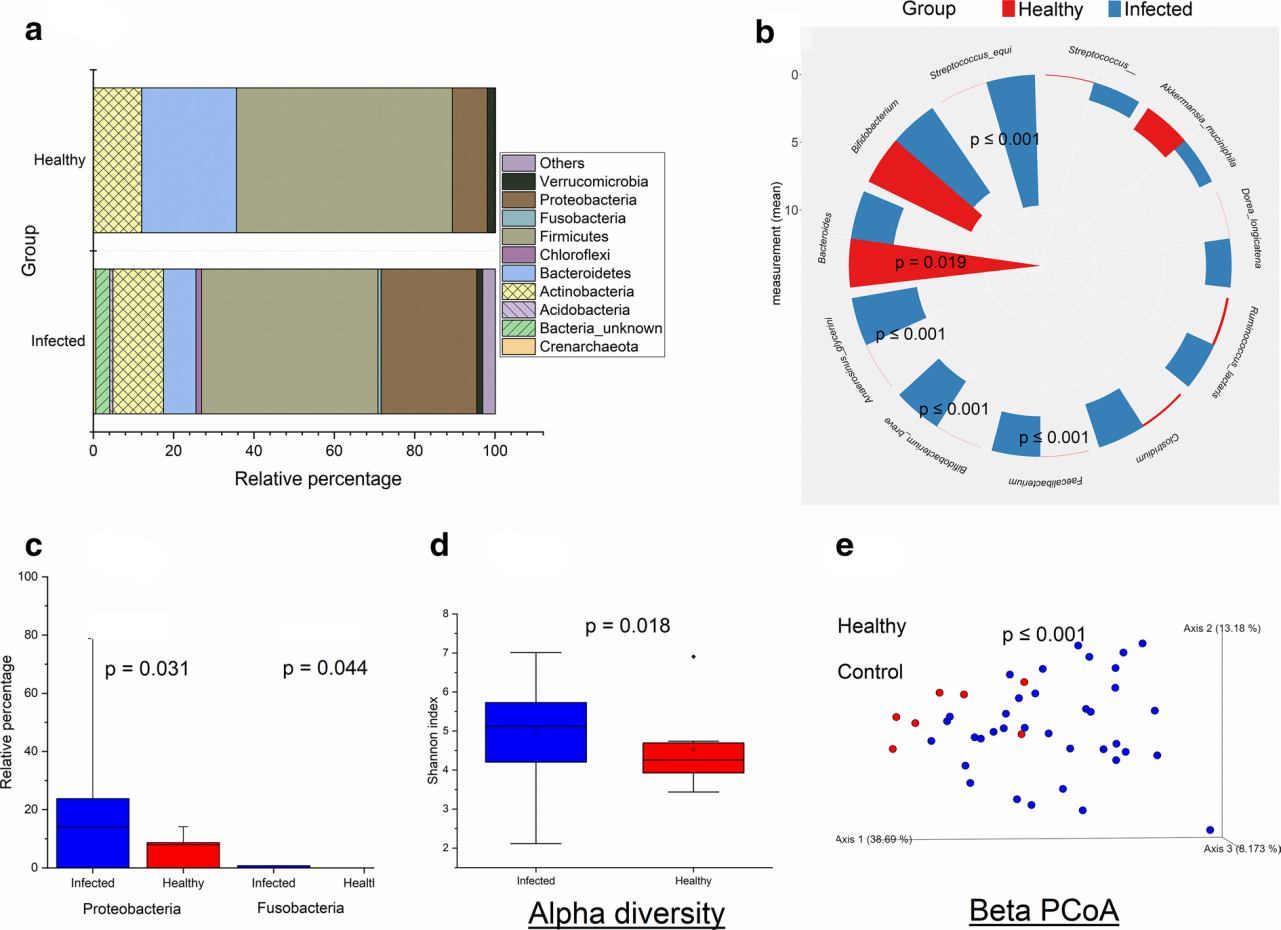
Microbiome comparison between infected and healthy children. Microbiome composition between the Infected (n = 39) and healthy control (n = 8) groups. **a** Relative percentage of the selected bacterial phyla. **b** Mean abundances of the bacterial species/genera. **c** Mean abundances of significantly different bacterial phyla. **d** Shannon-entropy of the study groups. **e** Beta diversity PCoA plots. Beta diversity comparisons were made using weighted unifrac index and statistical comparisons were made using PERMANOVA. p ≤ 0.05 is considered statistically significant and presented here

Taxonomic comparisons were made at the phylum, family, and species levels. The Mann–Whitney U test revealed that phylum Proteobacteria (p = 0.031) and Fusobacteria (p = 0.044) were statistically more abundant in the infected children compared with the healthy controls (Fig. 1a and c). At genus/species level, *Faecalibacterium*, *Bifidobacterium breve*, *Anaerosinus glycerini*, and *Streptococcus equi* were significantly higher (p ≤ 0.001), while *Bacteroides* were lower (p = 0.019) in the infected children compared with the control (Fig. 1b). Alpha diversity was calculated for the infected and healthy control groups using the Shannon diversity index, recorded as entropy scores, and elucidated in Fig. 1d. The entropy score increases as the richness and evenness of the species increase. Alpha diversity analysis showed that infected children had higher entropy scores (Shannon index; p = 0.018) compared with the controls, indicating higher bacterial abundance in the infected children. To describe the inter-individual variations between groups, weighted_unifrac analysis was performed. The relative cluster pattern of the gut microbiome in infected and healthy children is elucidated as a beta diversity PCoA graph (Fig. 1e). Weighted_unifrac analysis revealed separation and clustering of samples along the PC1 axis, while species appeared to cluster along the PC2 axis. The PCoA plot showed a distinct clustering pattern (PERMANOVA p ≤ 0.001) between the infected group and healthy controls.

### Microbiome comparison among gastroenteritis severity‐index, age, and gender groups

Children with moderate and severe gastroenteritis, ranked according to the Vesikari index, were compared. ‎Similarly, children were also compared based on their vomiting and diarrhea indexes (vomiting/diarrhea frequency vs. duration in days). No significant differences were observed among the indexed groups for microbiome diversity, richness, and taxonomy (Additional file 1: Figures S1, S2, and S3). Comparisons were also made for participants’ gender (male vs. female) and age groups (infants, toddler, and pre-School) groups. No statistical differences were observed among the age groups (Additional file 1: Figure S4). When comparisons were made between genders, only Phyla Fusobacteria and Verrucomicrobia were higher (p ≤ 0.01) in the females compared with the males. All other comparisons including alpha, and beta diversity, and genus/species level comparisons were non-significant between the two genders (Additional file 1: Figure S5).

### Microbiome comparison among Rotavirus vaccine (RVV) dosages groups

**Fig. 2:**
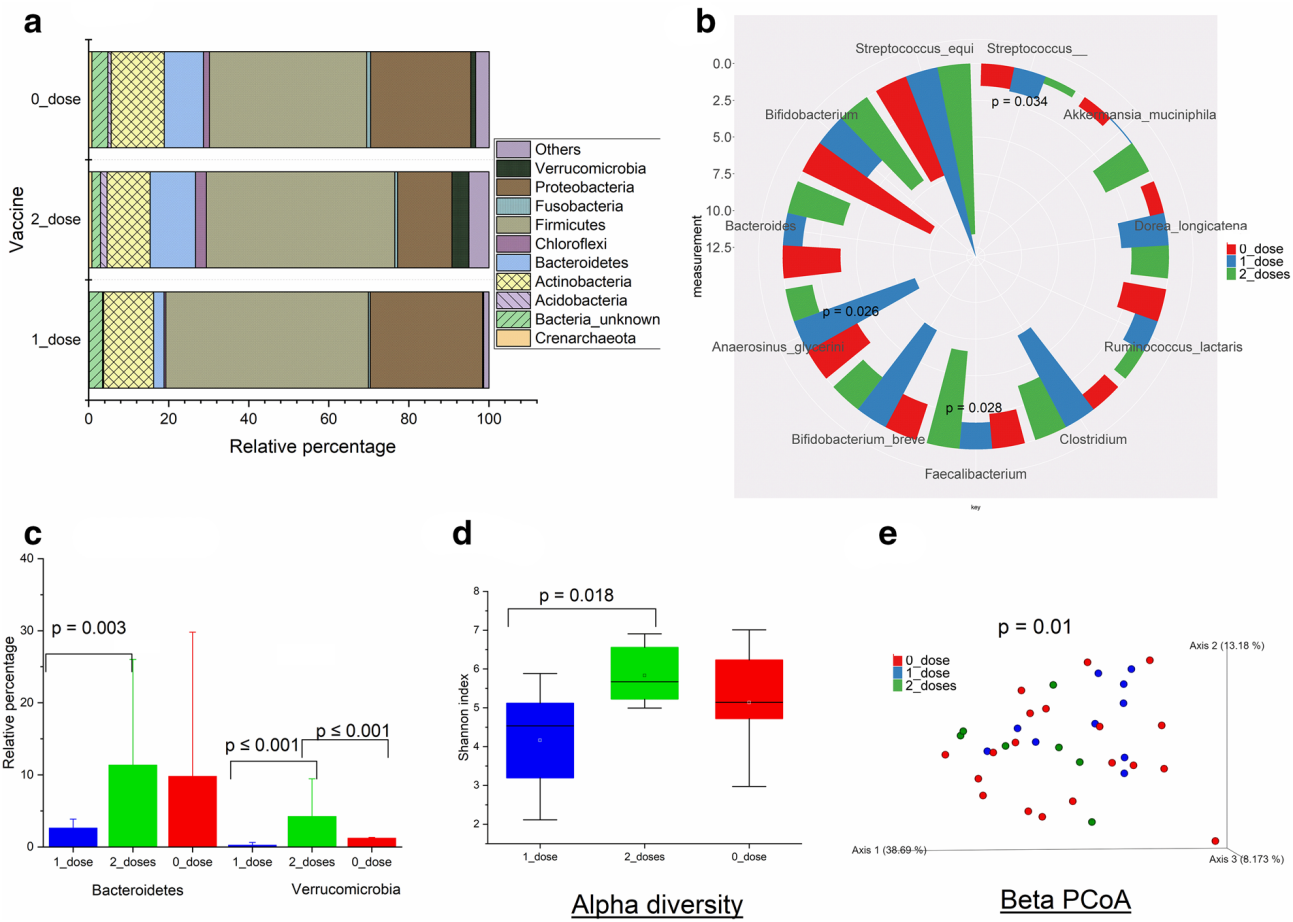
Microbiome comparison among Rotavirus vaccine (RVV) dosages groups. Microbiome composition among rotavirus vaccine 1_dose (n = 11), 2_doses (n = 7), and 0_dose (n = 21) groups. **a** Relative percentage of the selected bacterial phyla. **b** Mean abundances of the bacterial species/genera. Statistical comparisons were made using Kruskal Wallis test. **c** Mean abundances of significantly different bacterial phyla. Statistical comparisons were made using Kruskal Wallis test. **d** presents Shannon-entropy of the study groups. Statistical comparisons were made using Kruskal Wallis test. **e** Beta diversity PCoA plots. Beta diversity comparisons were made using weighted unifrac index and statistical comparisons were made using PERMANOVA. p ≤ 0.05 is considered statistically significant and presented here

Comparisons were made between children who were not vaccinated and the children with single and two vaccine shots. Children with two RVV doses had higher Bacteroidetes (p = 0.003) and Verrucomicrobia (p ≤ 0.001) phyla compared with the other groups (Fig. 2a, c). Family *Enterobacteriaceae* was significantly lower in children who received two RVV doses than the other groups. Similarly, at the genus/species level, *Faecalibacterium prausnitzii* (p = 0.028) was higher in the children who received two RVV doses than the other groups. However, genus *Streptococcus* (p = 0.034) and species *Anaerosinus glycerini* (p = 0.026) were higher in the children who received a single RVV dose compared with the other two groups (Fig. 2b). Shannon entropy analysis revealed that microbiome richness and evenness were higher (Shannon index; p = 0.018) in children who received two doses than the one dose group (Fig. 2d). Similarly, the beta diversity PCoA plot showed a distinct clustering pattern (PERMANOVA p = 0.01) between the 1_dose and 2_dose groups (Fig. 2e).

### Microbiome comparison among Rotavirus genotypes

Comparisons of microbiome were also made between different RV genotypes. No significant differences were observed between different genotype groups (Additional file 1: Figure S6).

### Correlation analysis

Spearman correlation analysis was made between quantitative clinical features and selected microbial taxa (Additional file 1: Figure S7). The analysis revealed few clinical features that significantly correlated with microbial taxa. Genus *Streptococcus* was negatively associated (r = − 0.52; p ≤ 0.001) with fever. Similarly, *Anaerosinus glycerini* was negatively associated (r = − 0.40; p ≤ 0.01) with dehydration. Genus *Bacteroides* was positively associated (r = 0.42; p ≤ 0.01) with age.

## Discussion

We compared the microbiome composition among thirty-nine RV positive children admitted at PEC, HMC, Doha, Qatar, and eight healthy controls. Previously, few studies have examined the microbiota of children with rotavirus, with a specific focus on RVV [2–4, 12, 13]. However, literature on microbiome analysis of RV gastroenteritis and comparison among different genotypes is scarce. This study indicated differences in microbiome diversity and taxonomic composition between RV-infected children and healthy controls. Similarly, significant differences were reported between vaccinated and non-vaccinated children. Particularly, the differences were more prominent between the 1_dose and 2_dose groups. No differences were observed among different RV genotypes. This may be attributed to the high variation in the sample size of RV genotypes.

We observed that both taxonomic composition and gut microbiome diversity were altered in the children with RV infection. Rotavirus infection was positively correlated with increased proportions of Proteobacteria and Fusobacteria. However, the most prevalent bacterial phyla, Firmicutes and Bacteroidetes, were not statistically different between the groups, albeit their higher abundance in the healthy controls. Such lack of significance may partly be due to higher inter-individual differences, resulting in high standard deviation. Similar observations have been reported previously in RV infected infants from China [14]. The authors observed an increase in the abundance of Proteobacteria in RV infected children compared to the controls. More recently, Engevik et al. [15] showed that RV infectivity and virulence are dependent on microbiome changes, particularly by elevated Proteobacteria and a decrease in abundance of Firmicutes. Gram-negative bacteria, especially Proteobacteria and Fusobacteria, can interfere with specific innate immune responses and cause severe gastritis expressing flagella or toxigenic lipopolysaccharides (LPS) [16]. In our samples, phylum Proteobacteria could be resolved at the family level, *Enterobacteriaceae*, which was the most abundant bacterial family that accounted for a double increase in the infected ‎children relative to the controls. Ramani et al. suggest that several pathogenic members of the *Enterobacteriaceae* family can potentially affect RV infectivity by indirect mechanisms such as modulating innate immune response and integrity of the intestinal wall [17]. Notably, *Enterobacteriaceae* family members, *Enterobacter* and *Klebsiella*, were the main genera driving the prediction of symptomatic RV infections in neonates [17]. An increase in alpha diversity relative to healthy controls was observed in the RV infected children. This observation contradicts previous studies, which recorded a decrease [14, 15, 18] or no effect on alpha diversity [19]. However, the increase in alpha diversity in the infected population could be due to the non-convergence of the family *Enterobacteriaceae*, which accounted for about 16 % and could not be further resolved at the level of genus/species.

Many of the previous RV microbiome studies have focused on the role of microbiome in the efficacy and immunogenicity of vaccines [2–4, 12, 13]. A popular hypothesis suggests that microbiome diversity is positively associated with RVV efficacy and seroconversion [2–4, 6, 12, 13]. However, dissimilar findings have been reported on the diversity/richness and taxonomic association of the microbiome with the RVV seroconversion [20]. Here, we observed vaccine dose-dependent variations in microbiome diversity and the abundance of many bacterial taxa. Children who received two vaccine doses had higher alpha diversity. Furthermore, the 2_doses group also had more abundance of Bacteroidetes and Verrucomicrobia and a lower population of Proteobacteria. Previous RVV seroconversion studies from Ghana and Pakistan showed similar changes in Proteobacteria and Bacteroidetes [2, 4]. In the current study, we only had data on vaccine doses but didn’t collect data on vaccine efficacy that worth investigating in future studies. Furthermore, diet is an important contributor of microbiome changes. We had no records for participant’s dietary habits. The study participants belonged to sixteen nationalties, which would offer distinct diet, socio-economic status, living status. These factors, particularly, socioeconomic status and hygiene contribute significantly to the RV virulence and microbiome [13].

‎‎Regarding the other comparisons, no statistical differences in microbiome composition and diversity were observed when comparing groups based on the severity of gastroenteritis, age, gender groups, or RV genotypes. These observations are in contrast with the previously reported findings by Mathew et al. [18], who reported an increase in the population abundance of certain bacterial clades due to fever, diarrhea, and vomiting. Similarly, Li et al. [14] reported a decrease in Shannon entropy in infants suffering from RV-associated diarrhea and vomiting. Notably, Mathew et al. [18] suggested that the increase in diarrhea frequency was positively correlated with *Enterobacteria* and *Klebsiella*.

Studies describing viral infections and microbiome are limited and ‎contradictory. Our observations and previous reports endorse diverse roles for these microbiomes in RV infection and vaccination. Therefore, our results suggest that manipulating these microbes could be a beneficial approach to study vaccine efficacy. Indeed, these preliminary observations require subsequent experimentation observing pre- and post-vaccine microbiome. Future experiments are warranted for the mechanistic investigation of vaccine metagenomic. Furthermore, uniform sample sizes among study groups shall be desired in future studies.

## Supplementary Information

**Additional file 1: Figure S1.** Microbiome comparison among gastroenteritis severity groups arranged according to Vesikari index, Microbiome composition between severe (n = 31) and moderate (n = 8) gastroenteritis groups. (S1a) presents relative percentage of the selected bacterial phyla. (S1b) presents mean abundances of the bacterial species/genera. Statistical comparisons were made using Mann-Whitney U test. (S1c) presents Shannon-entropy of the study groups. Statistical comparisons were made using Mann-Whitney U test. (S1d) presents beta diversity PCoA plots. Beta diversity comparisons were made using weighted unifrac index and statistical comparisons were made using PERMANOVA. p ≤ 0.05 was considered statistically significant and presented here. **Figure S2.**Microbiome comparison among diarrhea severity-index groups. Microbiome composition among mild (n = 16), moderate (n = 13), and severe (n = 10) diarrhea groups. (S2a) presents relative percentage of the selected bacterial phyla. (S2b) presents mean abundances of the bacterial species/genera. Statistical comparisons were made using Kruskal Wallis test. (S2c) presents Shannon-entropy of the study groups. Statistical comparisons were made using Kruskal Wallis test. (S2d) presents beta diversity PCoA plots. Beta diversity comparisons were made using weighted unifrac index and statistical comparisons were made using PERMANOVA. P ≤ 0.05 was considered statistically significant and presented here. **Figure S3.** Microbiome comparison among vomiting severity-index groups. Microbiome composition among mild (n = 15), moderate (n = 11), and severe (n = 13) diarrhea groups. (S3a) presents relative percentage of the selected bacterial phyla. (S3b) presents mean abundances of the bacterial species/genera. Statistical comparisons were made using Kruskal Wallis test. (S3c) presents Shannon-entropy of the study groups. Statistical comparisons were made using Kruskal Wallis test. (S3d) presents beta diversity PCoA plots. Beta diversity comparisons were made using weighted unifrac index and statistical comparisons were made using PERMANOVA. P ≤ 0.05 was considered statistically significant and presented here. **Figure S4.** Microbiome comparison among age groups. Microbiome composition among infants (n = 13), toddlers (n = 23), and pre-School (n = 11) children. (S4a) presents relative percentage of the selected bacterial phyla. (S4b) presents mean abundances of the bacterial species/genera. Statistical comparisons were made using Kruskal Wallis test. (S4c) presents Shannon-entropy of the study groups. Statistical comparisons were made using Kruskal Wallis test. (S4d) presents beta diversity PCoA plots. Beta diversity comparisons were made using weighted unifrac index and statistical comparisons were made using PERMANOVA. P ≤ 0.05 was considered statistically significant and presented here. **Figure S5.** Microbiome comparison between gender. Microbiome composition among male (n = 28) and female (n = 19) children. (S5a) presents relative percentage of the selected bacterial phyla. (S5b) presents mean abundances of the bacterial species/genera. Statistical comparisons were made using Mann-Whitney U test. (2c) presents mean abundances of significantly different bacterial phyla. Statistical comparisons were made using Mann-Whitney U test. (S5d) presents Shannon-entropy of the study groups. Statistical comparisons were made using Mann-Whitney U test. (S5e) presents beta diversity PCoA plots. Beta diversity comparisons were made using weighted unifrac index and statistical comparisons were made using PERMANOVA. P ≤ 0.05 was considered statistically significant and presented here. **Figure S6.** Microbiome comparison among Rotavirus genotypes. Microbiome composition among rotavirus genotypes; G3P8 (n = 25), G1P8 (n = 6), G2P4 (n = 4), G3P4 (n = 2), and G9P8 (n = 2). (S6a) presents relative percentage of the selected bacterial phyla. (S6b) presents mean abundances of the bacterial species/genera. Statistical comparisons were made using Kruskal Wallis test. (S6c) presents Shannon-entropy of the study groups. Statistical comparisons were made using Kruskal Wallis test. (S6d) presents beta diversity PCoA plots. Beta diversity comparisons were made using weighted unifrac index and statistical comparisons were made using PERMANOVA. P ≤ 0.05 was considered statistically and significant presented here. **Figure S7.** Correlation between microbiome and quantitative clinical features of the study participants. Spearman’s correlation analysis was performed using R package “corrplot 0.84” (RStudio version 3.5.0). The color intensity and shape of ellipse indicate the strength of the correlation depicted as r-value. Asterisks in each box indicate the p-value; ** ≤ 0.001 and * ≤ 0.01.

## Abbreviations

RV: Rotavirus
RVV: Rotavirus vaccine
PERMANOVA: Permutational multivariate analysis of variance
0_dose: Non-vaccinated RVV group
1_dose: One RVV dose group
2_doses: Two doses RVV group
GALT: Gut-associated lymphoid tissue
GIT: Gastrointestinal tract
AGE: Acute gastroenteritis
QIIME2: Quantitative Insights Into Microbial Ecolog2
PCoA: Principal coordinates
