# Supplementary material for: Microbiome profiling of rotavirus infected children suffering from acute gastroenteritis
Source: Gut Pathog. 2021 Mar 29;13:21. doi: 10.1186/s13099-021-00411-x (PMC8005861; doi:10.1186/s13099-021-00411-x)
Supplement: Supplementary file 1 — Additional file 1: Figure S1. Microbiome comparison among gastroenteritis severity groups arranged according to Vesikari index, Microbiome composition between severe (n = 31) and moderate (n = 8) gastroenteritis groups. (S1a) presents relative percentage of the selected bacterial phyla. (S1b) presents mean abundances of the bacterial species/genera. Statistical comparisons were made using Mann-Whitney U test. (S1c) presents Shannon-entropy of the study groups. Statistical comparisons were made using Mann-Whitney U test. (S1d) presents beta diversity PCoA plots. Beta diversity comparisons were made using weighted unifrac index and statistical comparisons were made using PERMANOVA. p ≤ 0.05 was considered statistically significant and presented here. Figure S2.Microbiome comparison among diarrhea severity-index groups. Microbiome composition among mild (n = 16), moderate (n = 13), and severe (n = 10) diarrhea groups. (S2a) presents relative percentage of the selected bacterial phyla. (S2b) presents mean abundances of the bacterial species/genera. Statistical comparisons were made using Kruskal Wallis test. (S2c) presents Shannon-entropy of the study groups. Statistical comparisons were made using Kruskal Wallis test. (S2d) presents beta diversity PCoA plots. Beta diversity comparisons were made using weighted unifrac index and statistical comparisons were made using PERMANOVA. P ≤ 0.05 was considered statistically significant and presented here. Figure S3. Microbiome comparison among vomiting severity-index groups. Microbiome composition among mild (n = 15), moderate (n = 11), and severe (n = 13) diarrhea groups. (S3a) presents relative percentage of the selected bacterial phyla. (S3b) presents mean abundances of the bacterial species/genera. Statistical comparisons were made using Kruskal Wallis test. (S3c) presents Shannon-entropy of the study groups. Statistical comparisons were made using Kruskal Wallis test. (S3d) presents beta diversity PCoA plots. Beta diversit [file 13099_2021_411_MOESM1_ESM.docx]

**Figure S1: Microbiome comparison among** **gastroenteritis severity groups arranged according to Vesikari index**


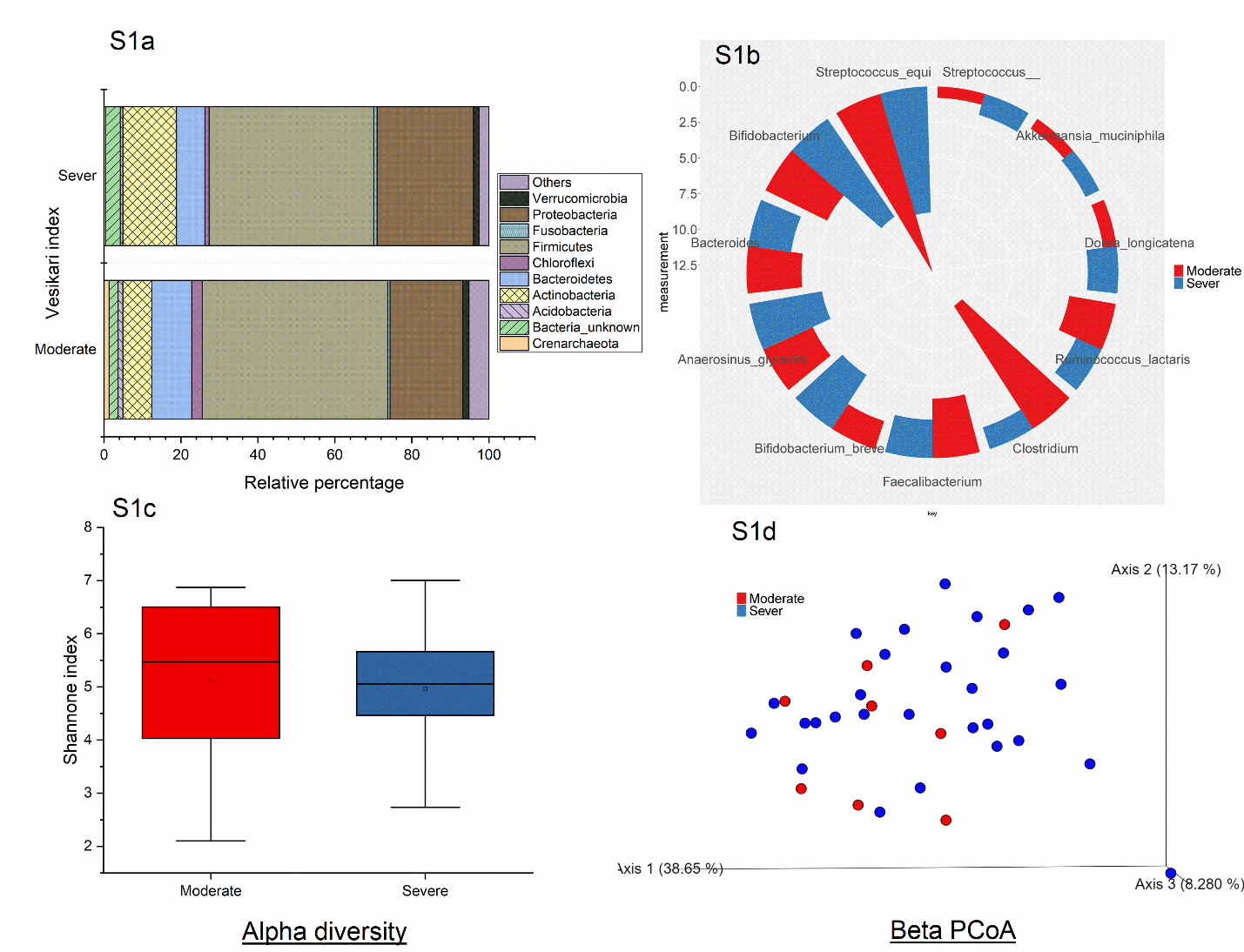


Microbiome composition between severe (n = 31) and moderate (n = 8) gastroenteritis groups. (S1a) presents relative percentage of the selected bacterial phyla. (S1b) presents mean abundances of the bacterial species/genera. Statistical comparisons were made using Mann-Whitney U test. (S1c) presents Shannon-entropy of the study groups. Statistical comparisons were made using Mann-Whitney U test. (S1d) presents beta diversity PCoA plots. Beta diversity comparisons were made using weighted unifrac index and statistical comparisons were made using PERMANOVA. P ≤ 0.05 was considered statistically significant and presented here.

**Figure S2: Microbiome comparison among diarrhea severity-index groups**


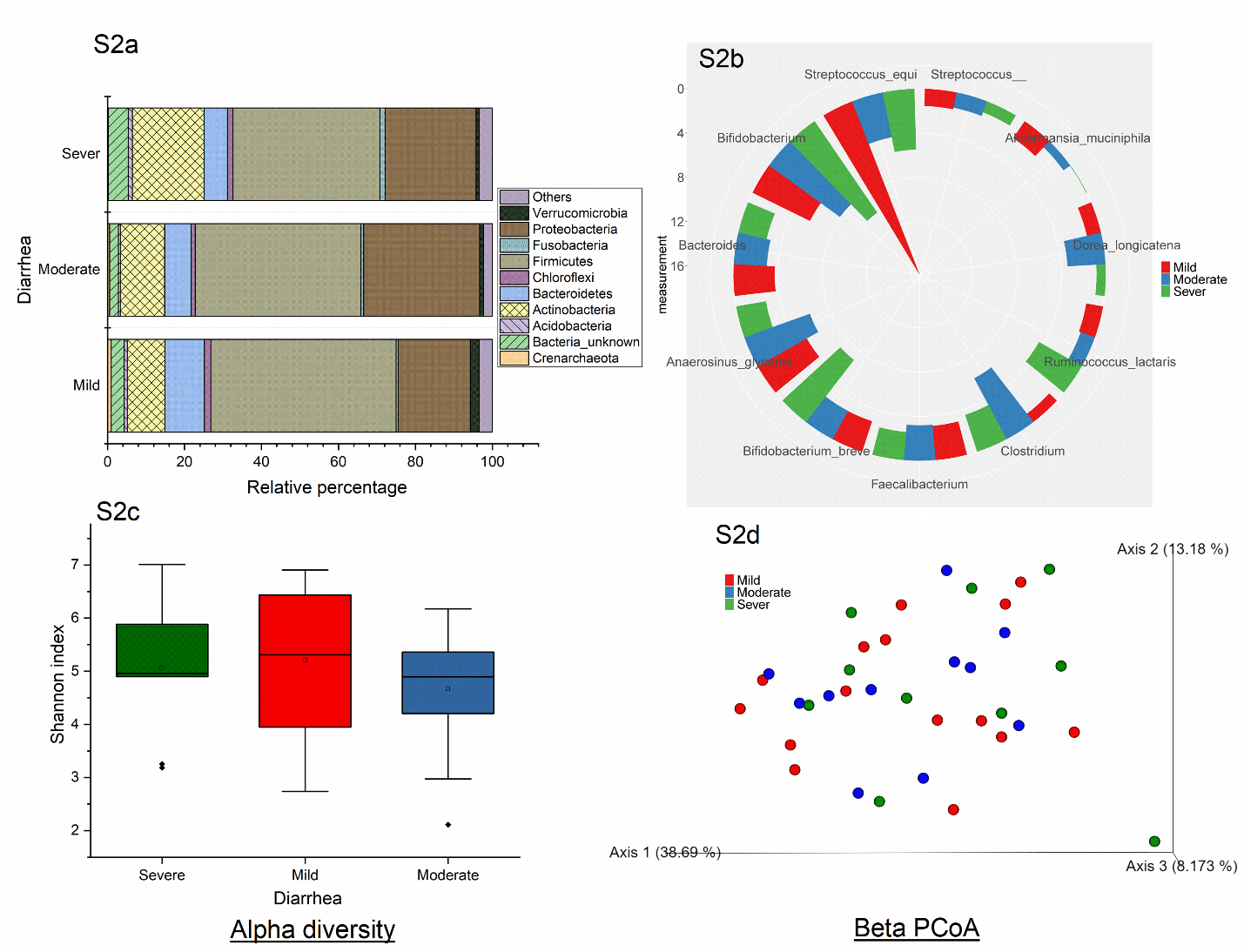


Microbiome composition among mild (n = 16), moderate (n = 13), and severe (n = 10) diarrhea groups. (S2a) presents relative percentage of the selected bacterial phyla. (S2b) presents mean abundances of the bacterial species/genera. Statistical comparisons were made using Kruskal Wallis test. (S2c) presents Shannon-entropy of the study groups. Statistical comparisons were made using Kruskal Wallis test. (S2d) presents beta diversity PCoA plots. Beta diversity comparisons were made using weighted unifrac index and statistical comparisons were made using PERMANOVA. P ≤ 0.05 was considered statistically significant and presented here.

**Figure S3: Microbiome comparison among vomiting severity-index groups**


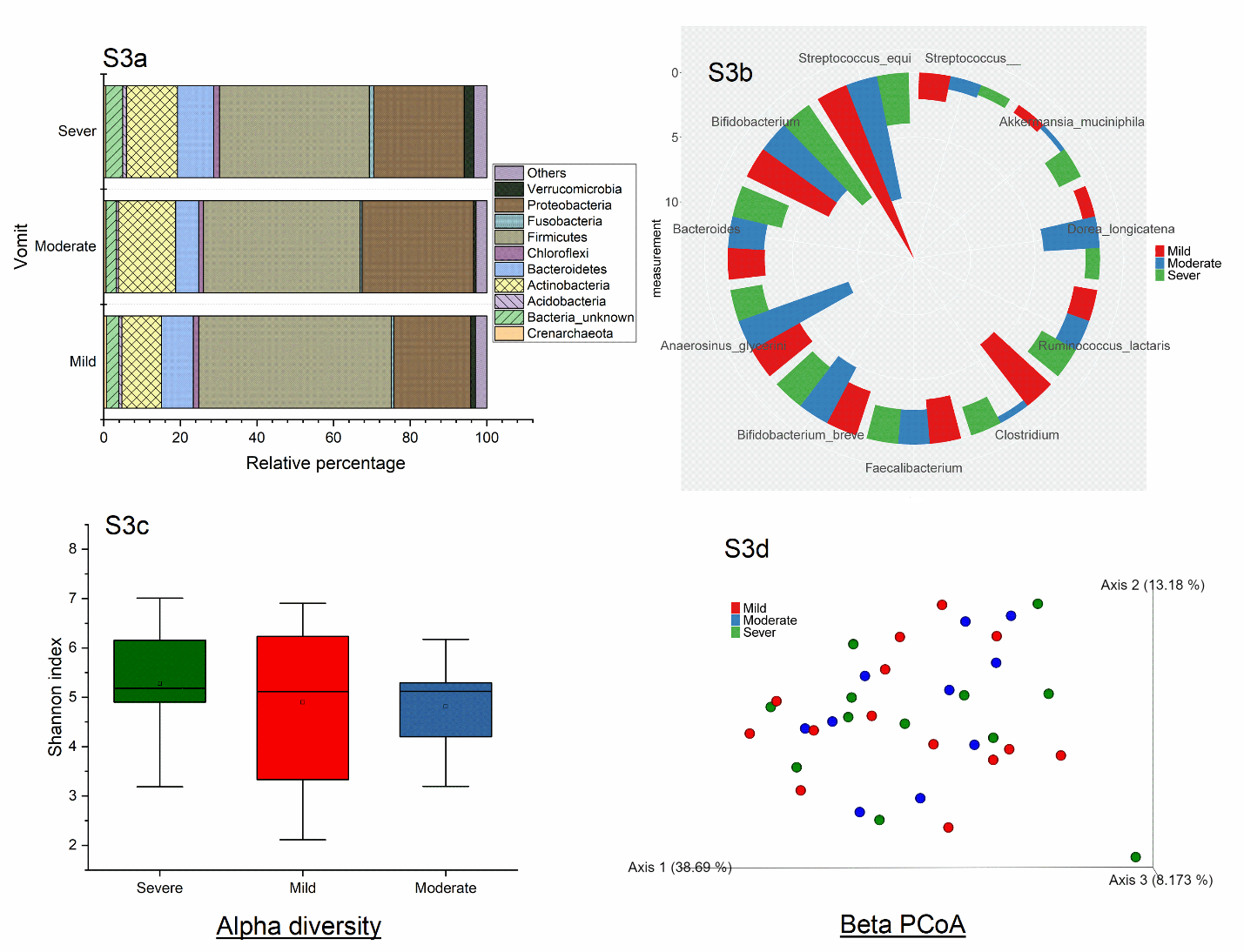


Microbiome composition among mild (n = 15), moderate (n = 11), and severe (n = 13) diarrhea groups. (S3a) presents relative percentage of the selected bacterial phyla. (S3b) presents mean abundances of the bacterial species/genera. Statistical comparisons were made using Kruskal Wallis test. (S3c) presents Shannon-entropy of the study groups. Statistical comparisons were made using Kruskal Wallis test. (S3d) presents beta diversity PCoA plots. Beta diversity comparisons were made using weighted unifrac index and statistical comparisons were made using PERMANOVA. P ≤ 0.05 was considered statistically significant and presented here.

**Figure S4: Microbiome comparison among age groups**


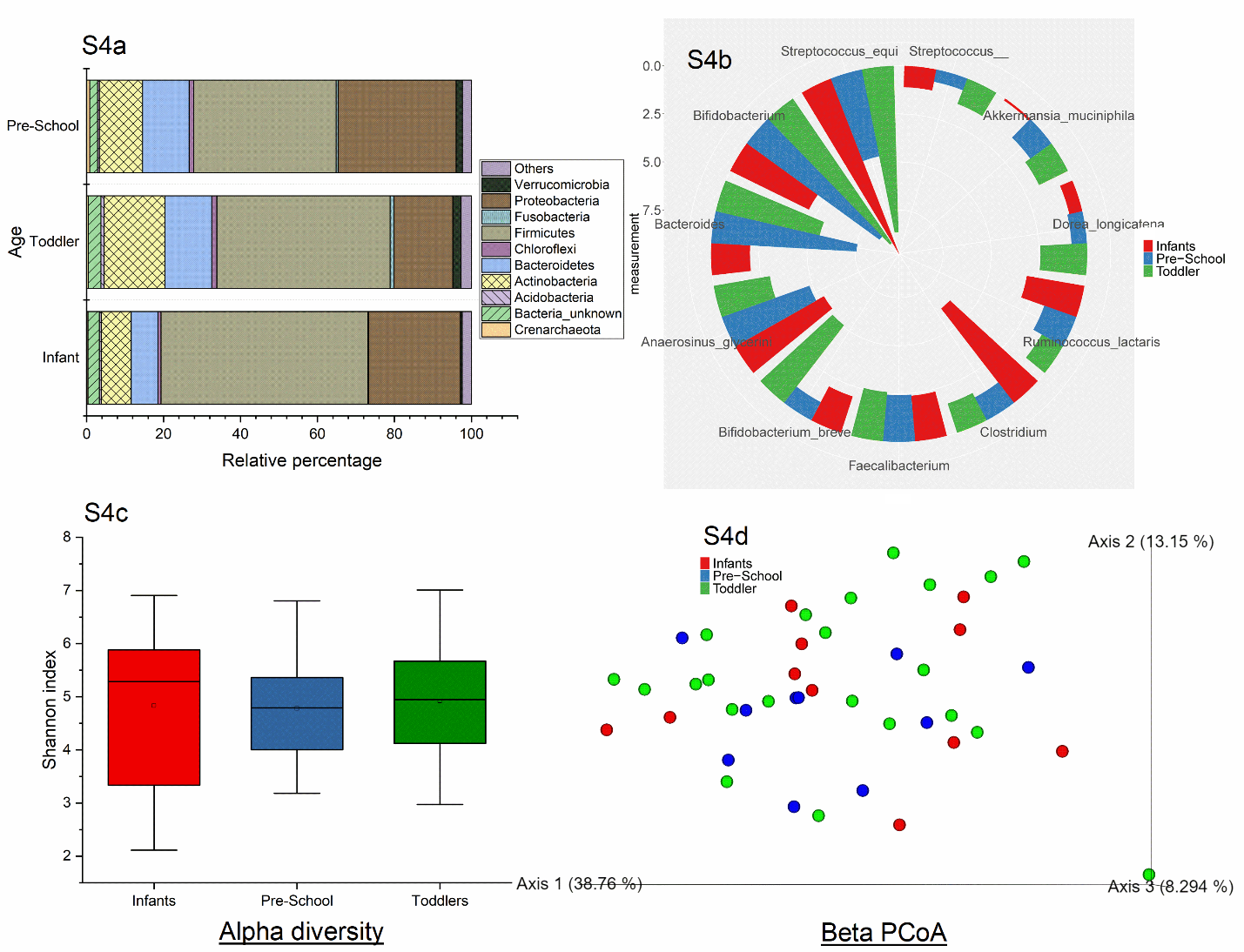


Microbiome composition among infants (n = 13), toddlers (n = 23), and pre-School (n = 11) children. (S4a) presents relative percentage of the selected bacterial phyla. (S4b) presents mean abundances of the bacterial species/genera. Statistical comparisons were made using Kruskal Wallis test. (S4c) presents Shannon-entropy of the study groups. Statistical comparisons were made using Kruskal Wallis test. (S4d) presents beta diversity PCoA plots. Beta diversity comparisons were made using weighted unifrac index and statistical comparisons were made using PERMANOVA. P ≤ 0.05 was considered statistically significant and presented here.

**Figure S5: Microbiome comparison between gender**


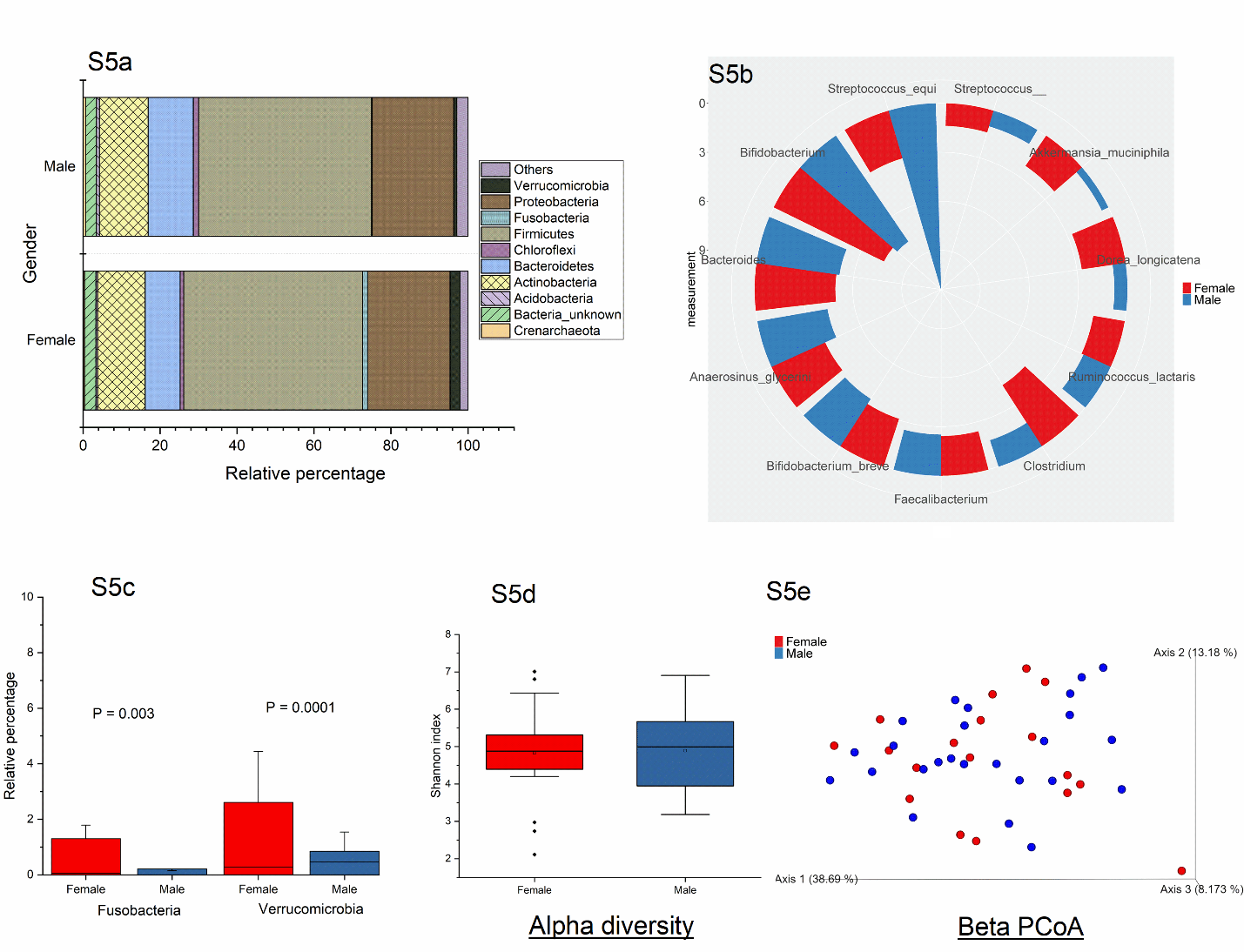


Microbiome composition among male (n = 28) and female (n = 19) children. (S5a) presents relative percentage of the selected bacterial phyla. (S5b) presents mean abundances of the bacterial species/genera. Statistical comparisons were made using Mann-Whitney U test. (2c) presents mean abundances of significantly different bacterial phyla. Statistical comparisons were made using Mann-Whitney U test. (S5d) presents Shannon-entropy of the study groups. Statistical comparisons were made using Mann-Whitney U test. (S5e) presents beta diversity PCoA plots. Beta diversity comparisons were made using weighted unifrac index and statistical comparisons were made using PERMANOVA. P ≤ 0.05 was considered statistically significant and presented here.

**Figure S6: Microbiome comparison among Rotavirus genotypes**


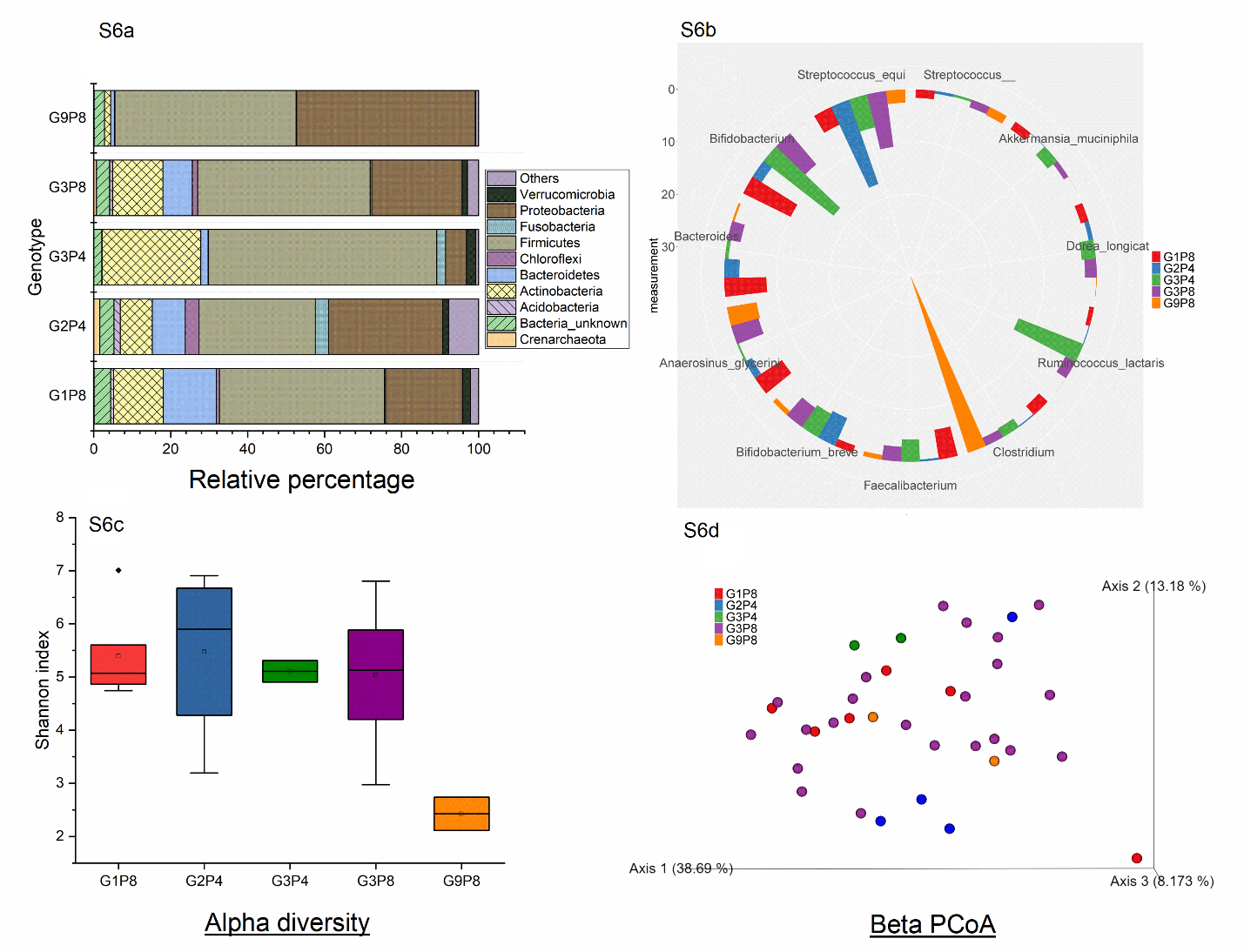


Microbiome composition among rotavirus genotypes; G3P8 (n = 25), G1P8 (n = 6), G2P4 (n = 4), G3P4 (n = 2), and G9P8 (n = 2). (S6a) presents relative percentage of the selected bacterial phyla. (S6b) presents mean abundances of the bacterial species/genera. Statistical comparisons were made using Kruskal Wallis test. (S6c) presents Shannon-entropy of the study groups. Statistical comparisons were made using Kruskal Wallis test. (S6d) presents beta diversity PCoA plots. Beta diversity comparisons were made using weighted unifrac index and statistical comparisons were made using PERMANOVA. P ≤ 0.05 was considered statistically and significant presented here.

**Figure S7: Correlation between microbiome and quantitative clinical features of the study participants**


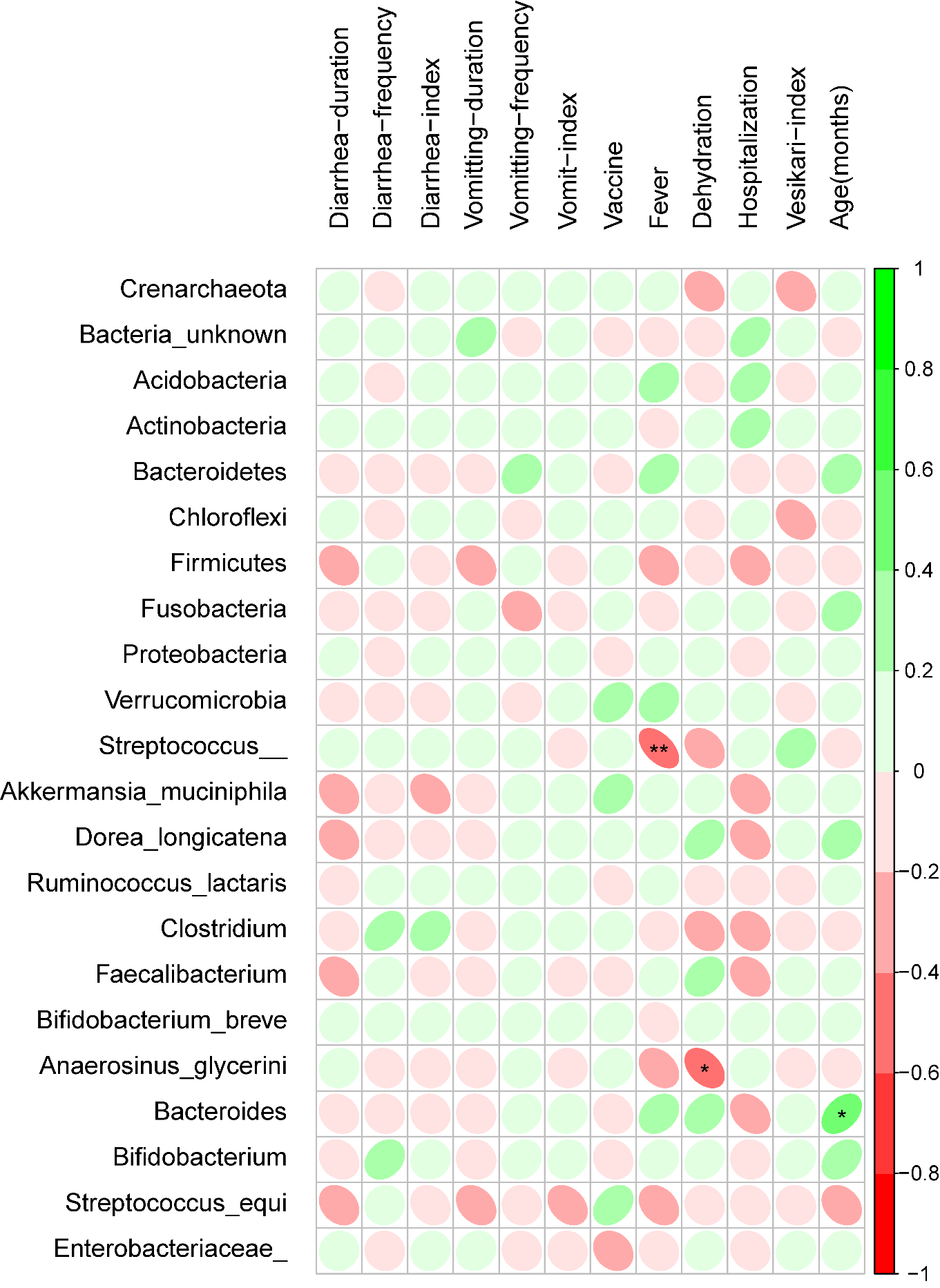


Spearman's correlation analysis was performed using R package "corrplot 0.84" (RStudio version 3.5.0). The color intensity and shape of ellipse indicate the strength of the correlation depicted as r-value. Asterisks in each box indicate the p-value; ** ≤ 0.001 and * ≤ 0.01.
